# Systematically reviewing the use of participatory methods in energy system modelling and planning literature

**DOI:** 10.1016/j.mex.2022.101862

**Published:** 2022-09-17

**Authors:** Connor McGookin, Brian Ó Gallachóir, Edmond Byrne

**Affiliations:** a School of Engineering, University College Cork, Ireland; b MaREI Centre, Environmental Research Institute, University College Cork, Ireland

**Keywords:** Energy and climate policy, Stakeholder engagement, Transdisciplinary research, Systematic literature review

## Abstract

This article outlines the systematic review process undertaken to identify what progress has been made on the integration of participatory methods into energy system modelling and planning. As an emergent field that combines technical / social sciences, it presented a couple of interesting challenges. Firstly, the issue of language emerged as there is a wide range of different terms that may be used to refer to both the involvement of stakeholders in research and energy system modelling and planning tools. This required careful consideration of the research questions and search criteria during the initial scoping exercise. On from this, a conceptual framing of what a meaningful stakeholder participation involves was developed to help define the criteria for inclusion in this study and assess the literature to date. Finally, in synthesizing the literature reviewed to provide an overview of the field, several creative data visualizations were produced.

• Systematic review process customized to identify literature covering the integration of participatory methods and energy system modelling and planning tools.
• Conceptual framework developed to define criteria for inclusion in the compiled database.

## Graphical abstract

**Figure:**
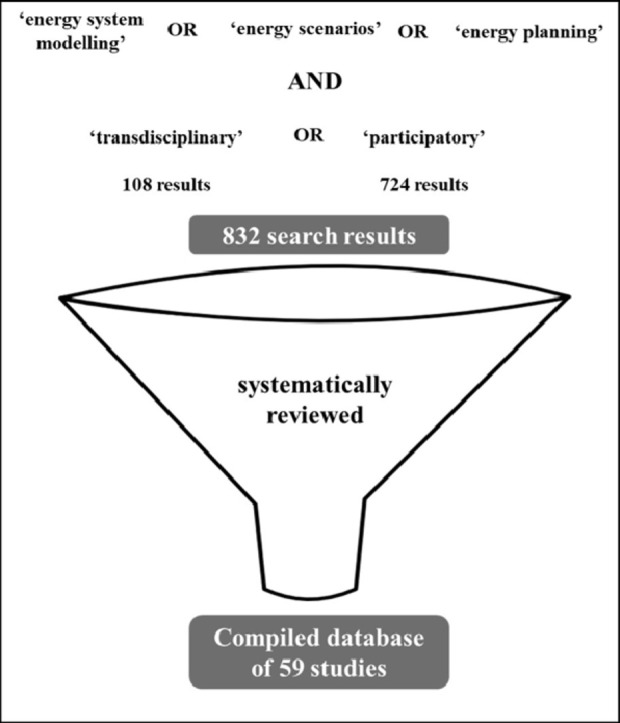

**SPECIFICATIONS TABLE**

**Table:** 

Subject Area:	Energy
More specific subject area:	Energy system modelling and planning
Method name:	Systematic literature review
Name and reference of original method:	• Grant & Booth (2009) “A typology of reviews: an analysis of 14 review types and associated methodologies” [1] • Onwuegbuzie et al. (2016) “Seven steps to a comprehensive literature review: A multimodal and cultural approach” [2]
Resource availability:	Compiled literature database provided in supplementary material

## Method details

Literature reviews help advance knowledge in a field by providing researchers with a synthesis of a body of knowledge that also highlights gaps in the literature and unaddressed research questions [1]. The use of a systematic literature review originates from medical and health research but is now quite common across a number of research fields [2]. It provides a useful means to compile a database of existing studies and to synthesize them in order to draw out implications for future research [3]. In comparison to a narrative review that seeks to provide a broad overview of a topic, a systematic review seeks to address a particular research question [4]. A defining feature of a systematic review is the use of a formal method that is transparent and replicable [5]. If the quality of the reporting is poor then this limits the reader's ability to assess the review, and thus reduces its credibility as a comprehensive assessment of the literature on a topic [6].

One source of confusion in this area is the fact that there are a wide range of review types with overlapping features [1], and the terminology used may carry different meanings across disciplines [7]. To identify the important elements of a review, Grant and Booth provide a useful framework called the SALSA framework - Search, AppraisaL, Synthesis and Analysis [1]. It breaks the process into four distinct elements: the initial search conducted, how inclusion and exclusion criteria were determined, the process of condensing the literature reviewed into useful findings and finally the variety of ways in which the literature is analysed. Another useful framework is the seven step process outlined by Onwuegbuzie et al., which includes the addition of reporting following the literature review [2]. Similarly, Mengist et al., expanding on Booth and Grant's framework, propose the PSALSAR approach with the addition of protocol at the beginning to define how the search will be conducted and reporting at the end [8].

The adopted four-step process outlined in this article for conducting a systematic review draws on these examples, and in particular the SALSA framework [1,5]. A key novelty of the present systematic review is the way in which progress to date of integrating participatory methods into energy system modelling and planning was determined. Firstly, a conceptual framework was developed to help understand what was required for a successful integration (see Section 2.1 in [9]). Secondly, who had been involved and how they participated in the research was assessed against established best practice in participatory research. It builds on the PSALSAR framework from Mengist et al. [8], giving particular attention to how the appraisal, synthesis and analysis steps were conducted.

In Part 1 the scope of the study is defined, beginning with the research questions that were formulated, an initial scoping search and literature selection criteria. Part 2 then involves the systematic review process to identify relevant studies. The means by which the compiled database was synthesized is detailed in Part 3, and finally Part 4 briefly introduces the reporting methods.

## Defining the scope

### Determine the scope

To understand how the scope of this study was determined it is useful to first explore why the pursuit of participatory methods within energy system modelling and planning has emerged as a research and policy priority in recent years. Many of the barriers to the development of renewable energy are non-technical challenges that are dynamic and context dependent. In the case of opposition to large-scale wind energy for example, existing research has shown a variety of conditions that shape public perception including physical, contextual, political, economic, social, local and personal aspects [10]. Transcending many of these issues are questions of procedural justice and trust in the actors when there is a perceived lack of public inclusion in the planning / decision-making process [11], [12], [13]. In the conventional approach to public policy decision-making, the goal of participation is often to educate the public on the merits of a particular project rather than involving them in an open democratic process [14]. As Sillak et al. note, the recent and growing interest in collaborative approaches to energy system planning has in a large part emanated as a response to the need to address this weakness in decision-making [15].

As outlined in McGookin et al., two key motivations behind the pursuit of participatory methods are; firstly to build a clearer picture of the energy system beyound technical or techno-economic representations, and secondly, to open up decision-making processes to a diverse range of stakeholders [9]. The latter of which is the primary focus of this review.

### Identify research questions

A useful first step in the systematic review process is to identify the research questions, which will ultimately define the scope of the study. A good research question will help guide decision-making when choosing search terms and defining the criteria for literature selection [5], as well as outlining the aim of the review [4].

The central research question posed was to what extent has the participatory process in energy system modelling and planning to date facilitated an open and transparent discussion on the best path forward. The following five research questions were taken to address this, as well as offer insights into the merits and challenges associated with different approaches, which may support further development in the field:

1. What stakeholders have been engaged in energy system modelling and planning? Moreover, to what extent has this involved engaging stakeholders outside of energy related fields?
2. To what extent has this involved a collaborative process as opposed to simply a consultation?
3. How have the qualitative outputs from stakeholder engagement been translated for use in quantitative energy system models or assessment tools?
4. What are the challenges and benefits of taking a participatory approach?
5. From the current body of literature, are there existing examples of best practice?

### Identify search terms

There were two key terms to identify: the search string for ‘participatory methods’ and for ‘energy system modelling and planning’. Both represent quite broad fields and understandings, so it was important to conduct an initial scoping search to identify the most commonly used keywords, as well as exploring potential variations in spelling. Table 1 provides a list of the terms tried in each case, along with a brief summary of the findings. The process involved firstly identifying terms synonymous with ‘energy system modelling’ in combination with the search term ‘participatory’, and then exploring variations of participatory with all three of the terms previously identified. A full outline of the search strings and number of results is provided in Appendix A, and an outline of what was defined as a relevant study is detailed in the following Section Define selection criteria and Table 2.

**Table 1:** Range of different search terms explored.

Terms Explored	Included/Excluded	Summary
Climate change mitigation	Excluded	Studies tended to deal with perceptions of climate change or adaptation planning
Energy modelling	Excluded	Large number of studies outside of the scope of this review involving; water management, health or urban planning
Energy system analysis	Excluded	Large number of studies outside of the scope of this review involving; life-cycle assessment, demand side management or urban planning
Energy system modelling	Included	Number of relevant studies found
Energy system model	Excluded	No additional relevant studies to those appearing under ‘energy system modelling’
Energy planning	Included	Number of relevant studies found
Energy scenarios	Included	Number of relevant studies found
Energy transition	Excluded	No additional relevant studies to those appearing under ‘energy system modelling’, ‘energy planning’ or ‘energy scenarios’
Engagement	Excluded	Large number of studies outside of the scope of this review, missing practical examples
Participatory	Included	Number of relevant studies found
Stakeholder participation	Excluded	Large number of studies outside of the scope of this review, missing practical examples
Stakeholder engagement	Excluded	No additional relevant studies to those appearing under ‘participatory’
Stakeholder dialogue	Excluded	No additional relevant studies to those appearing under ‘participatory’
Transdisciplinary	Included	Small number of relevant studies found

**Table 2:** Criteria for systematically selecting literature to be reviewed.

Included	Work published in the English language
	Studies involving stakeholder preferences, perceptions or opinions being established through some form of engagement, e.g. interviews, workshops, or meetings
	The stakeholder participation provided insights that informed the research and was not purely in the interest of data collection or awareness raising
	The output(s) of the stakeholder engagement were used as input(s) for qualitative or quantitative energy system analysis, i.e. the participatory process took place before the analysis was carried out
Excluded	Conference proceedings
	Studies without specific focus on a decision-making process as part of energy system modelling or planning
	Studies solely dealing with public attitude surveys toward a particular piece of existing infrastructure or new technology
	Papers noting the importance of stakeholder engagement as part of a methodology or concept but not providing an example of it in practice
	Stakeholder participation taking place after the quantitative analysis had been carried out

In the case of participatory methods, the two terms identified were ‘participatory’ and ‘transdisciplinary’, while for the energy system analysis the terms ‘energy system modelling’, ‘energy planning’ and ‘energy scenarios’ were chosen.

### Define selection criteria

The criteria used to determine what literature will be excluded from the compiled database is not always clear and is subject to bias based on the researcher's own framing [3]. Thus, it is important to clearly define the selection criteria (Table 2) and scope of the study (Determine the scope). This was found to be a particularly challenging issue in the present review due to the range of forms that stakeholder participation can take. As a result a conceptual framework was developed, as detailed in McGookin et al. [9]. It was based on the principle that stakeholder participation should seek to facilitate open and transparent discussion on key decision-making processes within energy system modelling and planning. We define meaningful participation as:

• Stakeholders being involved before technical analysis is conducted
• This involves some form of consultation and is not purely in the interest of information sharing or data collection

From this definition and the findings of the scoping search, the criteria shown in Table 2 were determined. Some common examples of studies that were initially included following review of titles and abstracts but subsequently determined to be out of scope (process discussed further in Systematic literature search) were:

• When the stakeholder engagement took place after the energy system analysis had already been conducted and thus had no bearing on it
• Studies solely involving public attitude surveys toward a particular piece of existing infrastructure.
• Participation was to provide data on household energy usage or preferences for emerging technologies but not to feed into a decision-making process on energy system configurations.

## Systematic literature search

The electronic database used to conduct a systematic review was SCOPUS. As outlined in Part 1.2, different search strings were identified for the Title, Abstract, Keywords field; “participatory” or “transdisciplinary” AND “energy system modelling” or “energy planning” or “energy scenarios”. The different combinations were searched separately as it was deemed useful to see what terms yielded the most results (Table 3). The original search was conducted on December 18^th^, 2018 and was kept up to date using the SCOPUS alert function until March 31^st^, 2021. It was not necessary to limit the temporal scope of the search as there were very few studies from before 2006 in the search results, which would be reflective of the fact that this is a new and emerging field. In addition to this, a snowballing technique was used to capture other relevant articles that had not appeared with the chosen keywords. This involved reviewing the bibliography of papers in the compiled database and also those that had cited articles in the compiled database [9].

**Table 3:** Number of papers found from each of the search strings used.

Search strings	Search results	Included
‘participatory’ & ‘energy system modelling’	86	19
‘transdisciplinary’ & ‘energy system modelling’	20	1
‘participatory’ & ‘energy planning’	386	9
‘transdisciplinary’ & ‘energy planning’	45	3
‘participatory’ & ‘energy scenarios’	154	13
‘transdisciplinary’ & ‘energy scenarios’	24	2
Snowballing technique		12
Total	715	59

The choice of software for data storing was Microsoft Excel, which is very useful for sorting and organising a systematic review [2]. The SCOPUS search results can be downloaded as a comma separate file, including information like the abstract, year of publication, source, etc. The first sorting step requires checking for duplicates and blank abstracts. Then the abstracts were reviewed using the criteria outlined in Table 2, to identify seventy-two articles for further review. However, having read the studies in full, a number were removed and excluded from the final compiled database. This was due to the fact that the studies lacked specific relevance. There were a number studies excluded from the detailed review as it became clear that the participatory element had taken place after a technical analysis had already been undertaken. In addition, studies that focused on understanding public attitude toward a specific piece of infrastructure such as the siting of a wind farm, overhead pylons or biomass plant were also excluded. This left forty-seven studies, with an additional twelve being added from those cited in the articles reviewed, giving a compiled database of fifty-nine studies.

## Synthesizing the literature

### Spatial and technology scale

Energy systems can exist at wide ranging variations in terms of spatial and technology scales. It is necessary to firstly categorize the studies by these two categories, as the different scales will require differences in approach. Spatially this ranged from studies looking at the national level (whole country) down to subnational (regions, cities, or towns). The technology focus varied from just a single technology (e.g. solar PV or wind energy) to a single or multiple modes (heat, transport or electricity), sectors (e.g. residential) and finally addressing the whole energy system. This list is provided in Table B.1 of Appendix B.

### Stakeholders engaged and level of participation

The first step was to identify which stakeholder groups had been involved in the research. This was done using stakeholder classifications adopted from the multi-actor perspective because it usefully divides actors across public organisations, private businesses and communities [9]. The three sectors are made up as follows: state (public agencies, government, etc.), market (firms, businesses, etc.) and community (households, individuals, etc.). Crosscutting all of these are non-profit organisations representing; state (universities, schools, etc.), market (cooperatives, associations, etc.) and community (charities, clubs, etc.). In addition, the number of participants was also captured in order to see what constitutes a typical (or desirable) amount. The full breakdown per study is provided in Tables B.2 and B.3 in Appendix B.

Secondly, a framework was needed to understand the level of participation. Based on the “Public Engagement Onion” developed by Welcome Trust [16] and a review of a transdisciplinary energy research project [17], the following framework was adopted;

• Informing – one-way flow of communication, usually for the purpose of awareness raising or educating, no opportunity for input into a decision-making process, participants cannot influence the outcome of the research.
• Consulting – two-way flow of communication, surveys, interviews, or workshops used to elicit stakeholder opinions, participants have opportunity to shape the research results but not the research questions or objectives.
• Collaborating – open and transparent communication throughout the process, participants given the opportunity to shape research questions and direction throughout the duration of the project.

### Methods used

Finally, the studies were grouped by the qualitative and quantitative methods used in order to draw out insights on the benefits or challenges of each, as well as common approaches for facilitating the integration of stakeholder input into quantitative energy system analysis. These are all listed in Table B.4 of Appendix B.

## Reporting & data visualization

The last step is to report on the findings of the literature review, which primarily involves publishing it in a peer-reviewed output such as a journal article. Within this, the use of creative visuals should be explored to communicate the synthesized literature. This may also prove useful for conferences or other oral presentations [2]. The comparative tables provided in Appendix B have all been condensed into visual representations for the accompanying journal article [9], this was done as follows:

• Figure 3: Number of studies at the different spatial and technology scales within the papers reviewed – This maps the share of the energy system covered in the studies on the x-axis based on expected share of total energy demand, e.g. a single technology focus (e.g. solar PV or bioenergy) taken to be less than 10%, or the whole energy system (100%). On the y-axis, there were three spatial scales from: national down to regional and then city/town. The data points are simply scaled based on the number of studies.
• Figure 4: Range of different stakeholders by share of papers that involved each group – This is a bar chart showing the share of studies involving each of the stakeholder groups identified.
• Figure 5: Level of stakeholder participation in the papers reviewed – This is a bar chart showing the number of studies that made use of each method alongside an established framework, Arnstein's ladder of citizen participation [18].
• Figure 6: Methods used in the papers reviewed by number of studies – This is a TreeMap chart showing what where the most commonly used methods.

## Declaration of competing interests

The authors declare that they have no known competing financial interests or personal relationships that could have appeared to influence the work reported in this paper.

## Appendix Supplementary materials

Image, application 1

Image, application 2
