# Supplementary material for: Systematically reviewing the use of participatory methods in energy system modelling and planning literature
Source: MethodsX. 2022 Sep 17;9:101862. doi: 10.1016/j.mex.2022.101862 (PMC9526136; doi:10.1016/j.mex.2022.101862)
Supplement: Supplementary file 1 [file mmc1.pdf]

Scopus search with strings input for Title, Abstract and Keyword, results limited to English and conference proceedings excluded

Search strings			No. of results
‘Climate change mitigation’	&	‘Participatory’	259
‘Energy modelling’	&	‘Participatory’	130
‘Energy system analysis’	&	‘Participatory’	253
‘Energy system modelling’	&	‘Participatory’	86
‘Energy system model’	&	‘Participatory’	168
‘Energy planning’	&	‘Participatory’	386
‘Energy scenarios’	&	‘Participatory’	154
‘Energy transition’	&	‘Participatory’	177
‘Engagement’	&	‘Energy system modelling’	97
‘Engagement’	&	‘Energy planning’	389
‘Engagement’	&	‘Energy scenarios’	148
‘Stakeholder participation’	&	‘Energy system modelling’	25
‘Stakeholder participation’	&	‘Energy planning’	207
‘Stakeholder participation’	&	‘Energy scenarios’	63
‘Stakeholder engagement’	&	‘Energy system modelling’	23
‘Stakeholder engagement’	&	‘Energy planning’	156
‘Stakeholder engagement’	&	‘Energy scenarios’	56
‘Stakeholder dialogue’	&	‘Energy system modelling’	9
‘Stakeholder dialogue’	&	‘Energy planning’	48
‘Stakeholder dialogue’	&	‘Energy scenarios’	19
‘Transdisciplinary’	&	‘Energy system modelling’	20
‘Transdisciplinary’	&	‘Energy planning’	45
‘Transdisciplinary’	&	‘Energy scenarios’	24
			2,942
