# Supplementary material for: Systematically reviewing the use of participatory methods in energy system modelling and planning literature
Source: MethodsX. 2022 Sep 17;9:101862. doi: 10.1016/j.mex.2022.101862 (PMC9526136; doi:10.1016/j.mex.2022.101862)
Supplement: Supplementary file 2 [file mmc2.pdf]

*Table A.1. List of papers reviewed in this study and their foci.*

| Title | Scale | | | Energy System Coverage | | | | | | | |
|---|---|---|---|---|---|---|---|---|---|---|---|
| | | Sub-national | | | Mode | | | Sector / Technology | | | |
| | National | Regional | City / Town | Whole System | Transport | Heating | Electricity | Housing | Bioenergy | Wind | Solar PV |
| A new hybrid approach for evaluating technology risks and opportunities in the energy transition in Ireland [1] | X | | | X | | | | | | | |
| Ambitious mitigation scenarios for Germany: A participatory approach [2] | X | | | X | | | | | | | |
| Benefits and challenges of participatory methods in qualitative energy scenario development [3] | X | | | X | | | | | | | |
| Effects of exemplar scenarios on public preferences for energy futures using the my2050 scenario-building tool [4] | X | | | X | | | | | | | |
| Long-term energy scenarios: Bridging the gap between socio-economic storylines and energy modelling [5] | X | | | X | | | | | | | |
| Low-carbon scenarios for Russia's energy system: A participative backcasting approach [6] | X | | | X | | | | | | | |
| Morocco's sustainable energy transition and the role of financing costs: A participatory electricity system modelling approach [7] | X | | | X | | | | | | | |
| Possible hydrogen transitions in the UK: Critical uncertainties and possible decision points [8] | X | | | X | | | | | | | |
| Reconciling qualitative storylines and quantitative descriptions: An iterative approach [9] | X | | | X | | | | | | | |
| Rethinking the role of scenarios: Participatory scripting of low-carbon scenarios for France [10] | X | | | X | | | | | | | |
| Sustainable energy futures: Methodological challenges in combining scenarios and participatory multi-criteria analysis [11] | X | | X | X | | | | | | | |
| Towards a sustainable hydrogen economy: A multi-criteria sustainability appraisal of competing hydrogen futures [12] | X | | | X | | | | | | | |
| Linking narratives and energy system modelling in transport scenarios: A participatory perspective from Denmark [13] | X | | | | X | | | | | | |
| Mitigation of CO2 emissions from the road passenger transport sector in Bahrain [14] | X | | | | X | | | | | | |

| | | | | | | | | | | | |
|---|---|---|---|---|---|---|---|---|---|---|---|
| New ways for the integrated appraisal of national energy scenarios: The case of renewable energy use in Austria [15] | X | | | | | X | X | | | | |
| A participatory multi-criteria approach for power generation and transmission planning [16] | X | | | | | | X | | | | |
| Collaborative energy visioning under conditions of illiberal democracy: results and recommendations from Ecuador [17] | X | | | | | | X | | | | |
| Efficiency versus equity in spatial siting of electricity generation: Citizen preferences in a serious board game in Switzerland [18] | X | | | | | | X | | | | |
| Imagine all these futures: On heterogeneous preferences and mental models in the German energy transition [19] | X | | | | | | X | | | | |
| Long-Term Electricity Scenarios for the MENA Region: Assessing the Preferences of Local Stakeholders Using Multi-Criteria Analyses [20] | X | | | | | | X | | | | |
| Models on the wrong track: Model-based electricity supply scenarios in Switzerland are not aligned with the perspectives of energy experts and the public [21] | X | | | | | | X | | | | |
| Perspectives of Informed Citizen Panel on Low-Carbon Electricity Portfolios in Switzerland and Longer-Term Evaluation of Informational Materials [22] | X | | | | | | X | | | | |
| Strategic and user-driven transition scenarios: Toward a low carbon society, encompassing the issues of sustainability and societal equity in Japan [23] | X | | | | | | X | | | | |
| Integrated decision-making about housing, energy and wellbeing: A qualitative system dynamics model [24] | X | | | | | | | X | | | |
| Participatory system dynamics modelling for housing, energy and wellbeing interactions [25] | X | | | | | | | X | | | |
| Austria's wind energy potential – A participatory modelling approach to assess socio-political and market acceptance [26] | X | | | | | | | | | X | |
| A transdisciplinary modelling framework for the participatory design of dynamic adaptive policy pathways [27] | X | | | | | | | | | | X |
| A methodological approach for holistic energy planning using the living lab concept: The case of the prefecture of Karditsa [28] | | X | | X | | | | | | | |
| Combining local preferences with multi-criteria decision analysis and linear optimization to develop feasible energy concepts in small communities [29] | | X | | X | | | | | | | |
| Transdisciplinary research for supporting environmental management [30] | | X | | X | | | | | | | |
| Using role play to explore energy perceptions in the United States and United Kingdom [31] | | X | | X | | | | | | | |
| Using scenario visioning and participatory system dynamics modelling to investigate the future: Lessons from Minnesota 2050 [32] | | X | | X | | | | | | | |
| Collaborative backcasting for transport policy scenario building [33] | | X | | | X | | | | | | |

| | | | | | | | | | | | |
|---|---|---|---|---|---|---|---|---|---|---|---|
| Achieving transparency and robustness of regional energy scenarios by using morphological fields in inter- and transdisciplinary project groups [34] | | X | | | | X | X | | | | |
| Balanced renewable energy scenarios: a method for making spatial decisions despite insufficient data, illustrated by a case study of the Vorderland-Feldkirch Region, Vorarlberg, Austria [35] | | X | | | | X | X | | | | |
| Developing transdisciplinary approaches to community energy transitions: An island case study [36] | | X | | | | X | X | | | | |
| Decision support framework for developing regional energy strategies [37] | | X | | | | | X | | | | |
| Informed public choices for low-carbon electricity portfolios using a computer decision tool [38] | | X | | | | | X | | | | |
| Methodology for Microgrid/Smart Farm Systems: Case of Study Applied to Indigenous Mapuche Communities [39] | | X | | | | | X | | | | |
| Regional energy planning through SWOT analysis and strategic planning tools: Impact on renewables development [40] | | X | | | | | X | | | | |
| Understanding energy-related regimes: A participatory approach from central Australia [41] | | X | | | | | | X | | | |
| A spatial explicit scenario method to support participative regional land-use decisions regarding economic and ecological options of short rotation coppice (SRC) for renewable energy production on arable land: case study application for the Göttingen district, Germany [42] | | X | | | | | | | X | | |
| Applying the Sustainability Science Principles of the Göttingen Approach to Initiate Renewable Energy Solutions in Three German Districts [43] | | X | | | | | | | X | | |
| Biomass feedstock and climate change in agroforestry systems: Participatory location and integration scenario analysis of biomass power facilities [44] | | X | | | | | | | X | | |
| Energy, forest, and indoor air pollution models for sagarmatha national park and buffer zone, Nepal [45] | | X | | | | | | | X | | |
| Engaging a rural agricultural community in sustainability indicators and future scenario identification: case of San Luis Valley [46] | | X | | | | | | | X | | X |
| Is enhanced biodiversity protection conflicting with ambitious bioenergy targets in eastern Finland? [47] | | X | | | | | | | X | | |
| Tweak, adapt, or transform: Policy scenarios in response to emerging bioenergy markets in the U.S. Corn Belt [48] | | X | | | | | | | X | | |
| Use of a participatory approach to develop a regional assessment tool for bioenergy production [49] | | X | | | | | | | X | | |
| An Interactive Planning Support Tool for Addressing Social Acceptance of Renewable Energy Projects in The Netherlands [50] | | | X | X | | | | | | | |

| | | | | | | | | | | | |
|---|---|---|---|---|---|---|---|---|---|---|---|
| Citizen-participatory scenario design methodology with future design approach: A case study of visioning of a low-carbon society in Suita City, Japan [51] | | | X | X | | | | | | | |
| Co-producing energy futures: impacts of participatory modelling [52] | | | X | X | | | | | | | |
| InSmart – A methodology for combining modelling with stakeholder input towards EU cities decarbonisation [53] | | | X | X | | | | | | | |
| Multicriteria decision support in local energy planning: An evaluation of alternative scenarios for the Sustainable Energy Action Plan [54] | | | X | X | | | | | | | |
| Societal implications of sustainable energy action plans: from energy modelling to stakeholder learning [55] | | | X | X | | | | | | | |
| Exploring scenarios for more sustainable heating: The case of Niš, Serbia [56] | | | X | | | X | | | | | |
| Supporting energy initiatives in small communities by linking visions with energy scenarios and multi-criteria assessment [57] | | | X | | | X | X | | | | |
| A methodology for community engagement in the introduction of renewable based smart microgrid [58] | | | X | | | | X | | | | |
| Transdisciplinary Evaluation of Energy Scenarios for a German Village Using Multi-Criteria Decision Analysis [59] | | | X | | | | | | X | | |

Note

1. “Participatory system dynamics modelling for housing, energy and wellbeing interactions” [25] details on participants taken from “Integrating GMB and games in the built environment” [60]
2. “Reconciling qualitative storylines and quantitative descriptions: an iterative approach” [9] modelling tool used taken from “Modelling generation and infrastructure requirements for transition pathways” [61]
3. “Societal implications of sustainable energy action plans: from energy modelling to stakeholder learning” [55] software used for analysis taken from “Strategic Energy Planning of Residential Buildings in a Smart City: A System Dynamics Approach” [62]
4. “Energy, Forest, and Indoor Air Pollution Models for Sagarmatha National Park and Buffer Zone, Nepal” [45] software used for analysis taken from “Experience With a Hard and Soft Participatory Modelling Framework for Social-ecological System Management in Mount Everest (Nepal) and K2 (Pakistan) Protected Areas” [63]

*Table A.2. Summary of the stakeholders involved with national studies.*

| Ref | State | | | | | | Market | | | | | | | Community | | | | | | | Number of participants |
|---|---|---|---|---|---|---|---|---|---|---|---|---|---|---|---|---|---|---|---|---|---|
| | Local | | Government / policy | | | | | | | | | | | | Formal Groups | | | | | | |
| | planning agency | unspecified | industry / labour | energy / infrastructure | other / unspecified | researchers / academia | energy | finance / bank | construction | agriculture / forestry | energy association | trade unions | unspecified | private individuals / citizens | environmental association | housing organisations | development organisation | youth organisation | unspecified | consumer association | |
| [1] | | | | x | | x | x | | | | | | x | | x | | | | | | 70 |
| [2] | x | | | | | | x | | | | | x | | | x | | | | x | x | 24 |
| [3] | | x | | x | | x | x | | | | x | | | | | | | | | x | NA |
| [4] | | | | | | | | | | | | | | x | | | | | | | 1800 |
| [5] | x | | | x | x | x | x | | | | | | x | | x | | | | | | 25 |
| [6] | | | | | | x | x | | | | | | x | | | | | | | | NA |
| [7] | | | | x | | x | x | | | | | | | | x | | | x | | | 25 |
| [8] | | | | | | x | x | | | | | | | | | | | | | | NA |
| [9] | | | | x | | | x | | | | | | | | x | | | | | | NA |
| [10] | | x | | x | | | x | x | | | x | x | x | | | | | | | x | 15 |
| [11] | | | x | x | x | x | x | | | x | x | | | | x | | | | | x | NA |
| [12] | | | | x | | x | x | | | | | | x | | x | | | | | | 40 |
| [13] | | | | x | | | x | | | | | | | | x | | | | | x | 24 |
| [14] | | | | x | x | | | | | | | | | | | | | | | | 40 |
| [15] | | | | x | | | x | | | | | | | | | | | | | | 25 |
| [16] | | | | | | x | | | | | | | | x | | | | | | | 380 |
| [17] | | | | x | x | x | x | x | | | | | x | | | | x | | x | | 32 |
| [18] | | | | | | | | | | | | | | x | | | | | | | 44 |
| [19] | | | | x | | | x | | | | x | | | | | | | | | x | 26 |
| [20] | | | | x | | x | x | | | | | | | | x | | | | | | 25 |
| [21] | | | | x | | x | x | | | | x | | | x | | | | | | | 167 |
| [22] | | | | | | | | | | | | | | x | | | | | | | 46 |
| [23] | | | | | | x | | | | | | | | x | | | | | | | 4148 |
| [24] | | x | | x | x | x | | | x | | | | | | | x | x | | | | 50 |
| [25] 1 | | x | | x | x | x | | | x | | | | | x | | x | | | | | NA |
| [26] | | | x | | x | | x | x | | | | | | | x | | | | | | 28 |
| [27] | | | | x | | | | | | | | | | | | | | | | | NA |

*Table A.3. Summary of the stakeholders involved with subnational studies.*

| Ref | State | | | | | | | | Market | | | | | | | | | | | | | Number of participants |
|---|---|---|---|---|---|---|---|---|---|---|---|---|---|---|---|---|---|---|---|---|---|---|
| | local | | | national | | | | | | | | | | | | formal group | | | | | | |
| | planning agency | government | energy / environment | energy / environmental | unspecified policymaker | labour / industry agencies | health / education | researchers / academia | energy industry | tourism | agriculture / forestry | construction | finance / bank | unspecified | private individuals / citizens | farmer's association | energy co-op / association | environmental association | religious institution | development organisation | unspecified | |
| [11] | | x | | | | | | x | x | | | x | | | x | | | x | | | | NA |
| [28] | | x | | | | x | | x | | | x | x | x | | | | | x | | x | | 17 |
| [29] | | x | x | | | | | | | | x | | | | x | | | | | | | 19 |
| [30] | x | | x | | | | | x | x | | | | x | | | x | | x | | | | NA |
| [31] | | | | | | | | | | | | | | | x | | | | | | | 83 |
| [32] | x | x | | x | x | | x | | | | x | | | | x | | | | x | | | NA |
| [33] | | | | | x | | | x | x | | | | | | x | | | | | | | NA |
| [34] | | | x | | | | | x | x | | | | x | x | | | | x | | | | 16 |
| [35] | | x | | | | | | x | x | | | | | | | | x | | | | | 30 |
| [36] | | | | | | | | | | | | | | | x | | | | | | | NA |
| [37] | | | | | | | | | | | | | | | x | | | | | | | 182 |
| [38] | | | | | | | | | | | | | | | x | | | | | | | 69 |
| [39] | | | | | | | | | | | x | | | | x | | | | | | | NA |
| [40] | | | | | | | | | x | x | x | | | | x | | | | | | x | 500 |
| [41] | | x | | | | | | | | | | | | | | | | x | | | x | 15 |
| [42] | x | | | | | | | | | | | | | | | x | | x | | | | 100 |
| [43] | | x | x | | | | | | | | x | | | | x | | | x | | | | NA |
| [44] | x | x | | | | | | x | x | | | | | x | | | | | | | | 105 |
| [45] | | | | x | | | | x | | x | | | | | x | | | | x | x | | NA |
| [46] | | x | | x | | | | | | | | | | | | x | | x | | | | NA |
| [47] | | | | x | | | x | x | x | x | x | | | x | | | | x | | | | 40 |
| [48] | | | | x | | | | | | | x | | | | | x | x | x | | | | 16 |
| [49] | | x | | | x | | | x | x | | x | | | | x | | | x | | | | NA |
| [50] | | x | | | | | | | | | | | | | x | | x | | | | | 35 |
| [51] | | | | | | | | | | | | | | | x | | | | | | | 446 |
| [52] | | x | | | | | | | | | | | | x | x | | | | x | | x | 15 |
| [53] | x | x | | | | | | x | x | | | | | x | | | | x | | | | NA |
| [54] | x | x | | | | | x | | x | | x | | | | x | | | | | | | NA |
| [55] | | x | | | | | | | x | | | | | | | | | | | | | NA |
| [56] | x | x | x | | | x | | x | x | | | | | | x | | | x | | | | NA |
| [57] | | x | | | | | | x | x | | | | | x | x | x | | x | | | | 29 |
| [58] | | | | | | | | | | | | | | | x | | x | | | | | 100 |
| [59] | | x | | | | | | | | | x | | | | x | | x | | | | | NA |

*Table A.4. Table of the qualitative and quantitative methods used in the studies reviewed.*

| | | Qualitative Analysis | | | | | | | | Quantitative Analysis | | | | | | |
|---|---|---|---|---|---|---|---|---|---|---|---|---|---|---|---|---|
| | | Eliciting Preferences | | | | Visioning / Mapping | | | | | | Energy System Models | | | | |
| | | Individual | | Group | | | | | | | | | | | | |
| | | survey /questionnaire | semi - structured interview | dialogue / deliberation | interactive display / serious game | scenario generation / backcasting | SWOT analysis | storytelling / visioning | cognitive mapping | multi-criteria decision analysis | sensitivity analysis | resource assessment | system dynamics modelling | agent based modelling | simulation tool | optimisation tool |
| National | [1] | | | x | | x | | | | | | | | | | x |
| | [2] | x | | | | | | x | | | | | | | | x |
| | [3] | x | | | | x | | x | | | | | | | | |
| | [4] | | | | x | | | | | | | | | | | |
| | [5] | | | | | x | | | | | | | | | | x |
| | [6] | | x | | | x | | | | | | | | | | |
| | [7] | | | | | x | | | | | | x | | | | |
| | [8] | | x | | | x | | | | | | | | | | x |
| | [9] | | x | | | x | | | | | | x | | | | |
| | [10] | x | | | | x | | | | | | | | | x | |
| | [11] | | x | | | x | | | | x | | | | | | |
| | [12] | | x | | | x | | | | x | | | | | | |
| | [13] | | | | | x | | | | | | | | | | x |
| | [14] | | x | | | x | | | | | | | | | x | |
| | [15] | | x | | | x | | | | x | | | | | | |
| | [16] | x | | | | | | | | x | | | | | | x |
| | [17] | | x | x | | | | x | | | | | | | | |
| | [18] | x | | x | x | | | | | | | | | | | |
| | [19] | | x | | | x | | | | | | | | | | |
| | [20] | x | | | | x | | | | x | | | | | x | |
| | [21] | x | | x | x | | | | | | | | | | | |
| | [22] | x | | | x | | | | | | | | | | | |
| | [23] | x | | | | | | | | | | | | | | x |
| | [24] | | x | | | | | x | x | | | | | | | |
| | [25] | | x | | | | | | x | | | | x | | | |
| | [26] | x | | | | x | | | | | | x | | | | |
| | [27] | | | x | | | | | | | | | | x | | |
| Subnational | [11] | | | | | x | | | | x | | x | | | | |
| | [28] | | | | | | x | | x | | | | | | | |
| | [29] | x | | | | | | | x | x | x | | | | | x |
| | [30] | | x | | | x | | | x | | | | | x | | |

| | | | | | | | | | | | | | | | | |
|---|---|---|---|---|---|---|---|---|---|---|---|---|---|---|---|---|
| | [31] | | | | x | | | | | | | | | | | |
| | [32] | | | | | x | | | x | | | | x | | | |
| | [33] | x | x | | | x | | | | | | | | | | |
| | [34] | | | x | x | x | | | | x | | | | | | |
| | [35] | | | | x | | | | | | | x | | | | |
| | [36] | x | x | x | | | | | | | | | | | | x |
| | [37] | x | | | x | | | | | | | | | | | |
| | [38] | x | | | x | | | | | | | | | | | |
| | [39] | x | x | | | | | | | | | x | | | | |
| | [40] | | | | | | x | | | | | x | | | | |
| | [41] | | x | | | x | | | | | | | | | | |
| | [42] | | | | | x | | | | x | | x | | | | |
| | [43] | | x | x | | | | x | | | | | | | | |
| | [44] | x | | | | | | | | x | | x | | | | |
| | [45] | | | | | x | | | x | | | | x | | | |
| | [46] | | x | x | | x | | | | | | x | | | | |
| | [47] | x | | | | | | | | x | | | | | | |
| | [48] | x | | | | x | | | | | | | | | | |
| | [49] | | x | | | | | | | x | | x | | | | |
| | [50] | | | x | x | | | | | | | | | | | |
| | [51] | x | | x | x | x | | x | x | | | | | | | |
| | [52] | | | | x | | | | | | | x | | | | |
| | [53] | | x | | | x | | | | x | | | | | | x |
| | [54] | x | | | | | | | | x | x | | | | | |
| | [55] | | | | | x | | | x | | | | x | | | |
| | [56] | | | | | x | x | x | | | | | | | x | |
| | [57] | | | | | x | | | | x | | x | | | | |
| | [58] | | x | x | | | | x | | | | | | | | |
| | [59] | | | x | | | | | | x | | x | | | | |

# References


1. Sharma, T., B.Ó. Gallachóir, and F. Rogan, *A new hybrid approach for evaluating technology risks and opportunities in the energy transition in Ireland.* Environmental Innovation and Societal Transitions, 2020.
2. Schmid, E. and B. Knopf, *Ambitious mitigation scenarios for Germany: A participatory approach.* Energy Policy, 2012. **51**: p. 662-672.
3. Ernst, A., et al., *Benefits and challenges of participatory methods in qualitative energy scenario development.* Technological Forecasting and Social Change, 2018. **127**: p. 245-257.
4. Demski, C., A. Spence, and N. Pidgeon, *Effects of exemplar scenarios on public preferences for energy futures using the my2050 scenario-building tool.* Nature Energy, 2017. **2**: p. 17027.
5. Fortes, P., et al., *Long-term energy scenarios: Bridging the gap between socio-economic storylines and energy modeling.* Technological Forecasting and Social Change, 2015. **91**: p. 161-178.
6. Sharmina, M., *Low-carbon scenarios for Russia's energy system: A participative backcasting approach.* Energy Policy, 2017. **104**: p. 303-315.
7. Schinko, T., et al., *Morocco's sustainable energy transition and the role of financing costs: a participatory electricity system modeling approach.* Energy, Sustainability and Society, 2019. **9**(1): p. 1.
8. McDowall, W. *Possible hydrogen transitions in the UK: Critical uncertainties and possible decision points*. 2012. Elsevier Ltd.
9. Robertson, E., et al., *Reconciling qualitative storylines and quantitative descriptions: an iterative approach.* Technological Forecasting and Social Change, 2017. **118**: p. 293-306.
10. Mathy, S., M. Fink, and R. Bibas, *Rethinking the role of scenarios: Participatory scripting of low-carbon scenarios for France.* Energy Policy, 2015. **77**: p. 176-190.

11. Kowalski, K., et al., *Sustainable energy futures: Methodological challenges in combining scenarios and participatory multi-criteria analysis.* European Journal of Operational Research, 2009. **197**(3): p. 1063-1074.
12. McDowall, W. and M. Eames, *Towards a sustainable hydrogen economy: a multi-criteria sustainability appraisal of competing hydrogen futures.* International Journal of Hydrogen Energy, 2007. **32**(18): p. 4611-4626.
13. Venturini, G., M. Hansen, and P.D. Andersen, *Linking narratives and energy system modelling in transport scenarios: A participatory perspective from Denmark.* Energy Research & Social Science, 2019. **52**: p. 204-220.
14. AlSabbagh, M., et al., *Mitigation of CO2 emissions from the road passenger transport sector in Bahrain.* Mitigation and Adaptation Strategies for Global Change, 2017. **22**(1): p. 99-119.
15. Madlener, R., K. Kowalski, and S. Stagl, *New ways for the integrated appraisal of national energy scenarios: the case of renewable energy use in Austria.* Energy Policy, 2007. **35**(12): p. 6060-6074.
16. Bertsch, V. and W. Fichtner, *A participatory multi-criteria approach for power generation and transmission planning.* Annals of Operations Research, 2016. **245**(1-2): p. 177-207.
17. Noboa, E., P. Upham, and H. Heinrichs, *Collaborative energy visioning under conditions of illiberal democracy: Results and recommendations from Ecuador.* Energy, Sustainability and Society, 2018. **8**(1).
18. Steinberger, F., T. Minder, and E. Trutnevyte, *Efficiency versus Equity in Spatial Siting of Electricity Generation: Citizen Preferences in a Serious Board Game in Switzerland.* Energies, 2020. **13**(18): p. 4961.
19. Schmid, E., et al., *Imagine all these futures: On heterogeneous preferences and mental models in the German energy transition.* Energy research & social science, 2017. **27**: p. 45-56.
20. Zelt, O., et al., *Long-term electricity scenarios for the MENA region: assessing the preferences of local stakeholders using multi-criteria analyses.* Energies, 2019. **12**(16): p. 3046.
21. Xexakis, G., et al., *Models on the wrong track: Model-based electricity supply scenarios in Switzerland are not aligned with the perspectives of energy experts and the public.* Renewable and Sustainable Energy Reviews, 2020. **134**: p. 110297.
22. Volken, S.P., G. Xexakis, and E. Trutnevyte, *Perspectives of informed citizen panel on low-carbon electricity portfolios in Switzerland and longer-term evaluation of informational materials.* Environmental science & technology, 2018. **52**(20): p. 11478-11489.
23. Chapman, A.J. and N.A. Pambudi, *Strategic and user-driven transition scenarios: Toward a low carbon society, encompassing the issues of sustainability and societal equity in Japan.* Journal of Cleaner Production, 2018. **172**: p. 1014-1024.
24. Macmillan, A., et al., *Integrated decision-making about housing, energy and wellbeing: A qualitative system dynamics model.* Environmental Health: A Global Access Science Source, 2016. **15**.
25. Eker, S., et al., *Participatory system dynamics modelling for housing, energy and wellbeing interactions.* Building Research and Information, 2018. **46**(7): p. 738-754.
26. Höltinger, S., et al., *Austria's wind energy potential–A participatory modeling approach to assess socio-political and market acceptance.* Energy policy, 2016. **98**: p. 49-61.
27. Michas, S., et al., *A transdisciplinary modeling framework for the participatory design of dynamic adaptive policy pathways.* Energy Policy, 2020. **139**: p. 111350.
28. Giannouli, I., et al., *A methodological approach for holistic energy planning using the living lab concept: The case of the prefecture of karditsa.* European Journal of Environmental Sciences, 2018. **8**(1): p. 14-22.
29. McKenna, R., et al., *Combining local preferences with multi-criteria decision analysis and linear optimization to develop feasible energy concepts in small communities.* European Journal of Operational Research, 2018. **268**(3): p. 1092-1110.
30. Düspohl, M., et al. *Transdisciplinary research for supporting environmental management*. 2012.
31. Thomas, M., et al., *Using role play to explore energy perceptions in the United States and United Kingdom.* Energy Research & Social Science, 2018. **45**: p. 363-373.
32. Olabisi, L.K.S., et al., *Using scenario visioning and participatory system dynamics modeling to investigate the future: Lessons from Minnesota 2050.* Sustainability, 2010. **2**(8): p. 2686-2706.
33. Soria-Lara, J.A. and D. Banister, *Collaborative backcasting for transport policy scenario building.* Futures, 2018. **95**: p. 11-21.
34. Droste-Franke, B., M. Voge, and A. Kanngießer, *Achieving transparency and robustness of regional energy scenarios by using morphological fields in inter-and transdisciplinary project groups.* Energy Strategy Reviews, 2020. **27**: p. 100430.
35. Nabielek, P., H. Dumke, and K. Weninger, *Balanced renewable energy scenarios: a method for making spatial decisions despite insufficient data, illustrated by a case study of the Vorderland-Feldkirch Region, Vorarlberg, Austria.* Energy, Sustainability and Society, 2018. **8**(1): p. 5.

36. Heaslip, E. and F. Fahy, *Developing transdisciplinary approaches to community energy transitions: An island case study.* Energy research & social science, 2018. **45**: p. 153-163.
37. Bessette, D.L., J. Arvai, and V. Campbell-Arvai, *Decision support framework for developing regional energy strategies.* Environmental science & technology, 2014. **48**(3): p. 1401-1408.
38. Mayer, L.A., W.n. Bruine de Bruin, and M.G. Morgan, *Informed public choices for low-carbon electricity portfolios using a computer decision tool.* Environmental science & technology, 2014. **48**(7): p. 3640-3648.
39. Vargas, C., et al. *Methodology for Microgrid/Smart Farm Systems: Case of Study Applied to Indigenous Mapuche Communities*. in *International Conference of ICT for Adapting Agriculture to Climate Change*. 2018. Springer.
40. Terrados, J., G. Almonacid, and L. Hontoria, *Regional energy planning through SWOT analysis and strategic planning tools. Impact on renewables development.* Renewable and Sustainable Energy Reviews, 2007. **11**(6): p. 1275-1287.
41. Foran, T., et al., *Understanding energy-related regimes: A participatory approach from central Australia.* Energy policy, 2016. **91**: p. 315-324.
42. Busch, G., *A spatial explicit scenario method to support participative regional land-use decisions regarding economic and ecological options of short rotation coppice (SRC) for renewable energy production on arable land: case study application for the Göttingen district, Germany.* Energy, Sustainability and Society, 2017. **7**(1).
43. Schmuck, P., M. Karpenstein-Machan, and A. Wüste, *Applying the sustainability science principles of the Göttingen approach to initiate renewable energy solutions in three German districts*, in *Sustainable Bioenergy Production-An Integrated Approach*. 2013, Springer. p. 319-338.
44. Jeong, J.S., *Biomass feedstock and climate change in agroforestry systems: Participatory location and integration scenario analysis of biomass power facilities.* Energies, 2018. **11**(6).
45. Salerno, F., et al., *Energy, forest, and indoor air pollution models for sagarmatha national park and buffer zone, Nepal.* Mountain Research and Development, 2010. **30**(2): p. 113-126.
46. Dubinsky, J., et al., *Engaging a rural agricultural community in sustainability indicators and future scenario identification: case of San Luis Valley.* Environment, Development and Sustainability, 2019. **21**(1): p. 79-93.
47. den Herder, M., et al., *Is enhanced biodiversity protection conflicting with ambitious bioenergy targets in eastern Finland?* Journal of Environmental Management, 2017. **187**: p. 54-62.
48. Atwell, R.C., L.A. Schulte, and L.M. Westphal, *Tweak, adapt, or transform: Policy scenarios in response to emerging bioenergy markets in the US corn belt.* Ecology and Society, 2011. **16**(1): p. 10.
49. Vaidya, A. and A.L. Mayer, *Use of a participatory approach to develop a regional assessment tool for bioenergy production.* Biomass and bioenergy, 2016. **94**: p. 1-11.
50. Flacke, J. and C. De Boer, *An interactive planning support tool for addressing social acceptance of renewable energy projects in the Netherlands.* ISPRS International Journal of Geo-Information, 2017. **6**(10).
51. Uwasu, M., et al., *Citizen-Participatory Scenario Design Methodology with Future Design Approach: A Case Study of Visioning of a Low-Carbon Society in Suita City, Japan.* Sustainability, 2020. **12**(11): p. 4746.
52. Krzywoszynska, A., et al., *Co-producing energy futures: impacts of participatory modelling.* Building Research and Information, 2016. **44**(7): p. 804-815.
53. Simoes, S., et al., *InSmart–A methodology for combining modelling with stakeholder input towards EU cities decarbonisation.* Journal of Cleaner Production, 2019. **231**: p. 428-445.
54. Marinakis, V., et al., *Multicriteria decision support in local energy planning: An evaluation of alternative scenarios for the Sustainable Energy Action Plan.* Omega (United Kingdom), 2017. **69**: p. 1-16.
55. Bernardo, G. and S. D'Alessandro, *Societal implications of sustainable energy action plans: from energy modelling to stakeholder learning.* Journal of Environmental Planning and Management, 2019. **62**(3): p. 399-423.
56. Zivkovic, M., et al., *Exploring scenarios for more sustainable heating: The case of Niš, Serbia.* Energy, 2016. **115**: p. 1758-1770.
57. Trutnevyte, E., M. Stauffacher, and R.W. Scholz, *Supporting energy initiatives in small communities by linking visions with energy scenarios and multi-criteria assessment.* Energy Policy, 2011. **39**(12): p. 7884-7895.
58. Alvial-Palavicino, C., et al., *A methodology for community engagement in the introduction of renewable based smart microgrid.* Energy for Sustainable Development, 2011. **15**(3): p. 314-323.
59. Schmuck, P., *Transdisciplinary evaluation of energy scenarios for a German village using multi-criteria decision analysis.* Sustainability, 2012. **4**(4): p. 604-629.
60. Carnohan, S., N. Zimmermann, and E. Rouwette. *Integrating GMB and games in the built environment*. in *http://www. systemdynamics. org/web. portal? A1223+ 0*. 2016. System Dynamics Society.

61. Barnacle, M., et al., *Modelling generation and infrastructure requirements for transition pathways.* Energy Policy, 2013. **52**: p. 60-75.
62. Caponio, G., et al., *Strategic energy planning of residential buildings in a smart city: a system dynamics approach.* International Journal of Engineering Business Management, 2015. **7**: p. 20.
63. Salerno, F., et al., *Experience With a Hard and Soft Participatory Modeling Framework for Social-ecological System Management in Mount Everest (Nepal) and K2 (Pakistan) Protected Areas.* Mountain Research and Development, 2010. **30**(2): p. 80-93.
